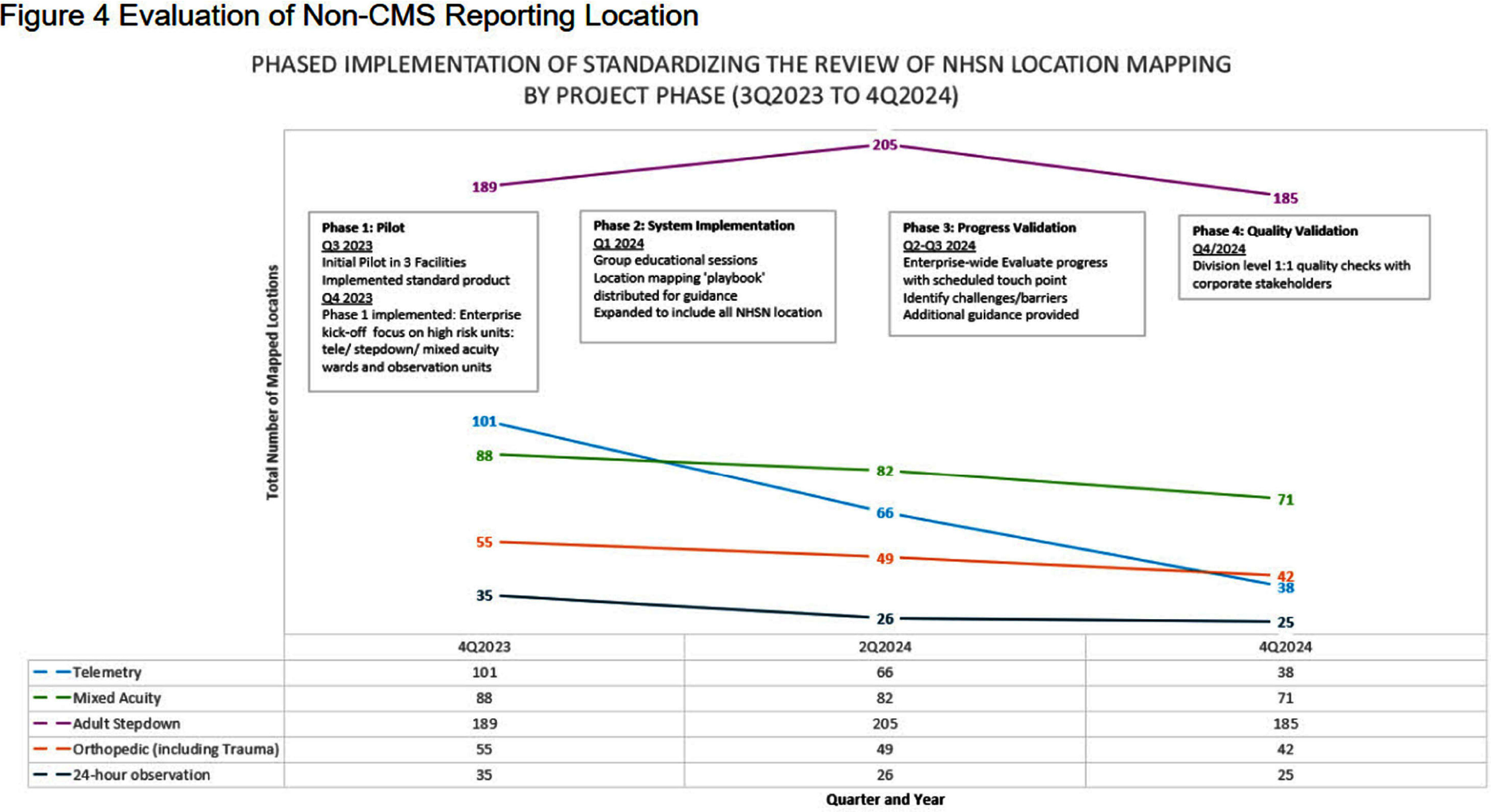# Tackling NHSN Location Mapping in a Large Healthcare System Utilizing a Robust Decision Support Tool

**DOI:** 10.1017/ash.2025.212

**Published:** 2025-09-24

**Authors:** Julia Moody, Rachel Long, Keetha Kratzer, Maribeth Coluni, Melissa Gallant, Danna Harmon

**Affiliations:** 1 HCA Healthcare; 2 HCA; 3 HCA Healthcare corporate ofice; 4 HCA Corporate ITG

## Abstract

**Background:** Hospitals experienced increased demand for acute care and specialty services during recovery following COVID-19 epidemics. Internal analysis identified potential inaccuracies in NHSN location unit designations across a large healthcare system with 2,249 mapped NHSN locations. Findings revealed inconsistencies in how location change decisions were determined mainly from the type of data applied. Facilities utilized finance data to determine NHSN location mapping creating limitations. NHSN locations defined by patient populations, associated with baseline risk adjustments to provide comparison performance and impacts CMS metrics. **Methods:** Decision Support Tool (DST) based on NHSN to evaluate patient populations for acuity, service line data indicating specialty services, financial billing codes, DRG and surgical or non-surgical populations. The DST outlines data elements for review when validating mapped locations. Each tab represents a unique data element to analyze as part of the NHSN decision algorithm (Figure 1). Unit level data aggregates in DST tabs (Figure 2). Implementation consisted of a pilot followed by a regionally phased implementation via coaching calls and follow-up touchpoints. Although all units were reviewed, specific activities focused on non-CMS reportable units such as telemetry, step-down, mixed acuity. As a part of the defined change control process facilities followed an internal standardized workflow to document changes, dates and reasons for change for historical reference (Figure 3). NHSN facility population changes were applied to vendor surveillance software utilized for CDA direct reporting to NHSN. System and facility internal record keeping promotes a standardized process for data validity, associated software maintenance and CMS reporting compliance. **Results:** The majority of changes were made to units mapped as telemetry with a 62% reduction overall. Figure 4 illustrates the non-CMS reporting locations with notable location mapping changes. Patients in an ‘observation’ status were found to be housed within any inpatient unit and required another data tool for analysis. Overall, the number of mapped 24-hour observation units is low (1.7%) across the healthcare system. **Conclusions:** The initiative standardized objective data and competency which elevated the trained infection preventionists on this topic. Admission orders offer telemetry for evaluation and treatment requiring continuous cardiac monitoring. NHSN definition is specific requiring 80% of unit patients to have a cardiac centered DRG/care and cardiac specialty treatment to meet telemetry definition. NHSN recommends at minimum annual mapping evaluation. As a large healthcare system, the DST analysis is managed continuously due to growing service lines, acquisitions and construction projects.

**Figure:**
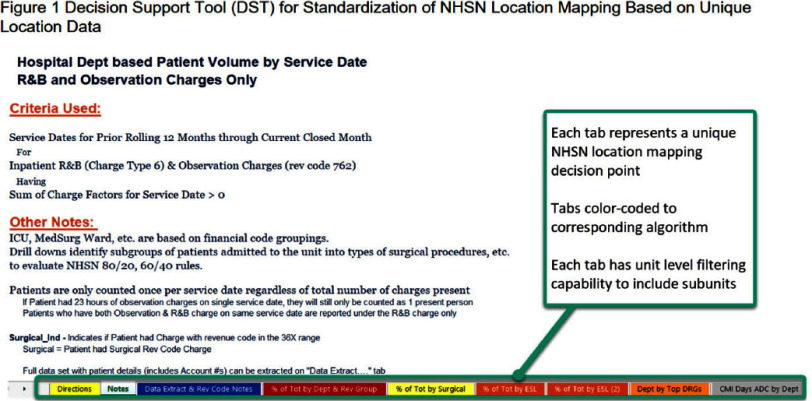

**Figure:**
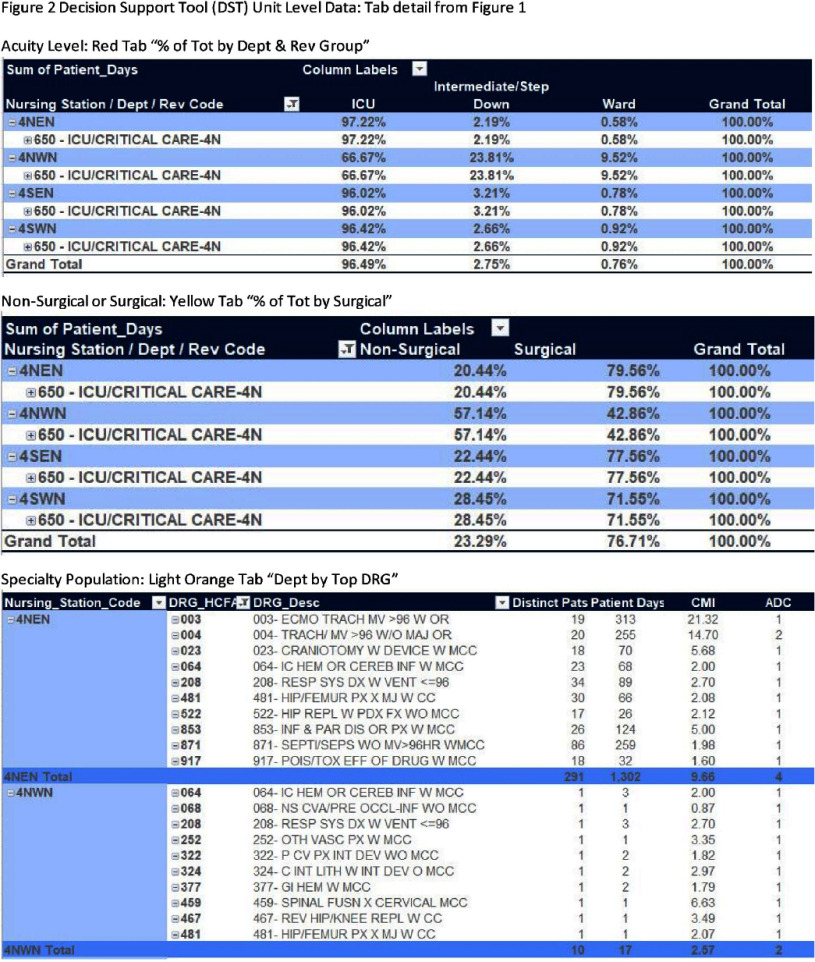

**Figure:**
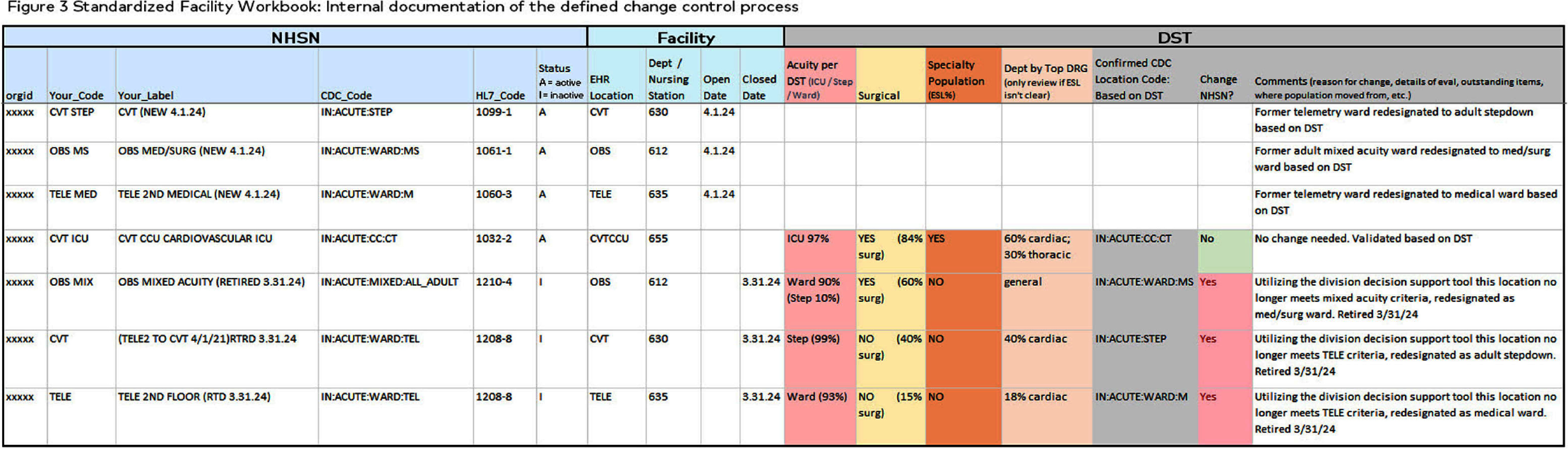

**Figure:**